# Supplementary figures and images for: Effects of Weather and Environmental Factors on the Seasonal Prevalence of Foodborne Viruses in Irrigation Waters in Gyeonggi Province, Korea
Source: Microorganisms. 2020 Aug 11;8(8):1224. doi: 10.3390/microorganisms8081224 (PMC7465913; doi:10.3390/microorganisms8081224)

Numbers of detected viruses

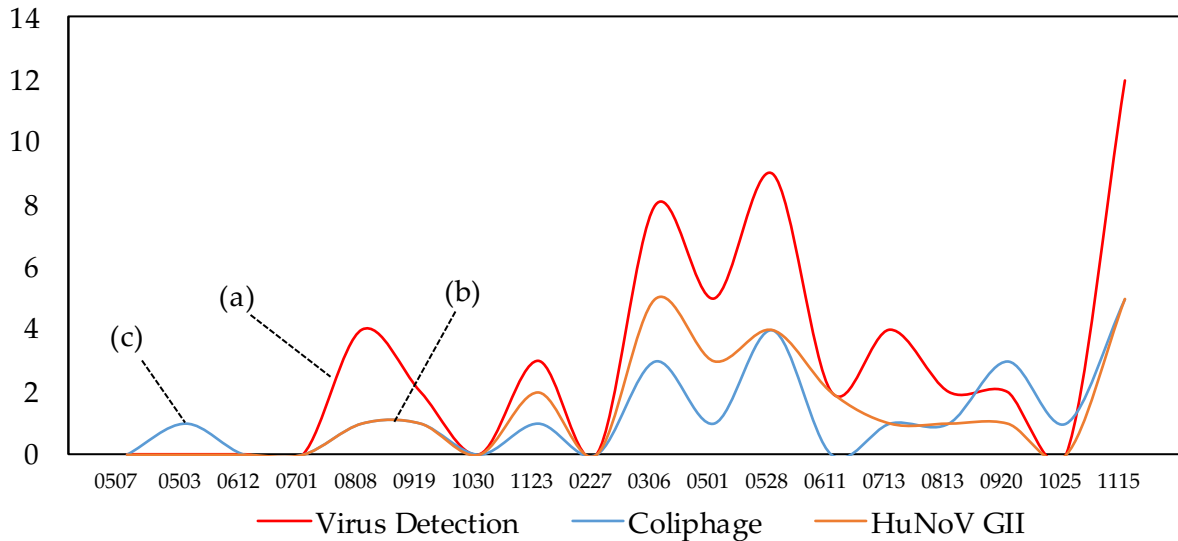

Supplement: Supplementary file 1 [file microorganisms-08-01224-s001.pdf]
